# The first complete plastome of *Chimonobambusa quadrangularis* (Fenzl) Makino: assembly, annotation and phylogenetic analysis

**DOI:** 10.1080/23802359.2021.1967808

**Published:** 2021-08-25

**Authors:** Fengming Ren, Liqiang Wang, Wei Zhuo, Xiaofu Zhu, Shenge Lu, Hongyan Huang, Dongliang Chen

**Affiliations:** a Research and Utilization on Characteristic Biological Resources of Sichuan and Chongqing Co-construction Lab, Chongqing Institute of Medicinal Plant Cultivation, Chongqing, China; b College of Pharmacy, Heze University, Heze, China; c Chongqing Shangyao Huiyuan Pharmaceutical Co. Ltd, Chongqing, China

**Keywords:** *Chimonobambusa quadrangularis*, plastome, phylogenetic analysis, Square Bamboo

## Abstract

*Chimonobambusa quadrangularis* (Fenzl) Makino is one of the ‘Square Bamboo’ due to its square-shaped culm. However, as an edible bamboo, there is no genomic information reported so far. In this study, we reported and characterized the first plastome of *C. quadrangularis* based on Illumina Hiseq sequencing. The plastome exhibited a typical angiosperm circular structure, containing four regions: large single-copy region (LSC: 83,125 bp), small single-copy region (SSC: 12,811 bp), and a pair of inverted repeat regions (IR: 21,802 bp). The plastome consisted of 139,540 bp in size, with 82 protein-coding genes, 39 tRNA genes, and eight rRNA genes. The total nucleotide composition consisted of 30.16% A, 30.97% T, 19.25% C, and 19.63% G. The G + C content of the whole plastome was 38.88%. Phylogenetic analysis based on the complete plastomes of six species indicated that *C. quadrangularis* was closed to *C. hejiangensis*. The plastome is helpful for studying the evolution of beneficial adaptations and developing bioremediation and biomedical science.

*Chimonobambusa quadrangularis* (Fenzl) Makino is an interesting and mystic group of bamboo. Some species of *Chimonobambusa* have quadrangular culms, or very tumid nodes, and their shoots appear during the colder season. The genus is distributed in areas with an altitude of 500–2600 m in China, Burma, Laos, Japan, and India. The species of the genus are able to endure cold, which can be introduced to high altitude and humid areas. Their shoots are seen as delicious food (Wen 1994).

*Chimonobambusa quadrangularis* is one of the most important species in this genus, which is originally cultivated in the provinces of Southwest China, especially in Guizhou and Chongqing (Inoue et al. 2019). The greatest value of the species is its edible shoots, which are considered both nutritionally and deliciously (Chen et al. 2019). In view of their taste and nutritional values, the bamboo shoots of the species are popular with consumers (Li et al. 2017). However, as an edible bamboo, there is no genomic information reported so far. The complete plastome is an excellent resource for studying the evolution of favorable traits and adaptations within a genus. In this study, we reported and characterized the first plastome of *C. quadrangularis* based on Illumina Hiseq sequencing.

Fresh leaves of *C. quadrangularis* were collected from Nanchuan, Chongqing, China (107°23′ E, 29°15′ N, 602 m). The voucher specimen was deposited in Chongqing Institute of Medicinal Plant Cultivation (accession number: CIMPC-RFM-20210302, Contact person: Fengming Ren; Email: 348080877@qq.com). Total genomic DNA was extracted by the kit method (Beijing Kinco Biological Company) following the manufacturer’s protocol. The purity and integrity of the DNA were analyzed by Nanodrop (Thermo Fisher Scientific) microvolume UV-Vis spectrophotometer and agarose gel electrophoresis. DNA libraries were constructed by using total genomic DNA, with an insert size of 350 bp. The libraries were sequenced by using HiSeq platform (Illumina, San Diego, CA). The raw data from the platform was removed low-quality reads and adapters by trimmomatic (version 0.35) with default paprameters (Bolger et al. 2014). The DNA fragments with a length of 150 bp were assembled using *de novo* assembly method by NOVOPlasty (version 4.1) with the default parameters (Nicolas et al. 2016), and the assembled plastome was annotated by CPGAVAS2 software with default parameters (http://47.96.249.172:16019/analyzer/home) (Shi et al. 2019). After manual check and adjustment, the annotated plastome was submitted to GenBank (MW928533).

The complete plastome of *C. quadrangularis* was 139,540 bp in length, which contained 82 protein-coding genes, 39 tRNA genes, and eight rRNA genes. The base composition of the plastome was estimated to be 30.16% A, 30.97% T, 19.25% C, 19.63% G, with a G + C content of 38.88%. The plastome exhibited a typical angiosperm circular structure, containing four regions: LSC was 83,125 bp, SSC was 12,811 bp, and IR was 21,802 bp. The G + C contents of each genomic region were 36.95% (LSC region), 33.22% (SSC region), and 44.22% (IR region).

To infer the phylogenetic placement of *C. quadrangularis*, a maximum-likelihood (ML) phylogenetic tree was constructed by using IQ-TREE (Nguyen et al. 2015) with 1000 bootstrap replicates (Figure 1). Plastome sequences of *C. quadrangularis* and other three *Chimonobambusa* species, including *C. sichuanensis* (MT941921), *C. tumidissinoda* (NC_036814) and *C. hejiangensis* (NC_053872), were used for reconstructing the ML tree. *Ananas comosus* (NC_026220) and *Typha angustifolia* (NC_050678) were set as outgroups. Before the reconstruction of the phylogenetic tree, all the six plastome sequences were aligned by MAFFT software with the parameter of ‘–auto’ (Standley and Daron 2013) under parameters of ‘-nt AUTO -m MFP -bb 1000 -bnni’. The result shown that all *Chimonobambusa* formed a monophy. And *C. quadrangularis* was the sister of *C. hejiangensis* (Figure 1). The complete plastome of *C. quadrangularis* is beneficial in studying the evolution of beneficial adaptations to aid in biomedical research and further phylogenetic research of *Chimonobambusa* and in the related genus.

**Figure 1.**
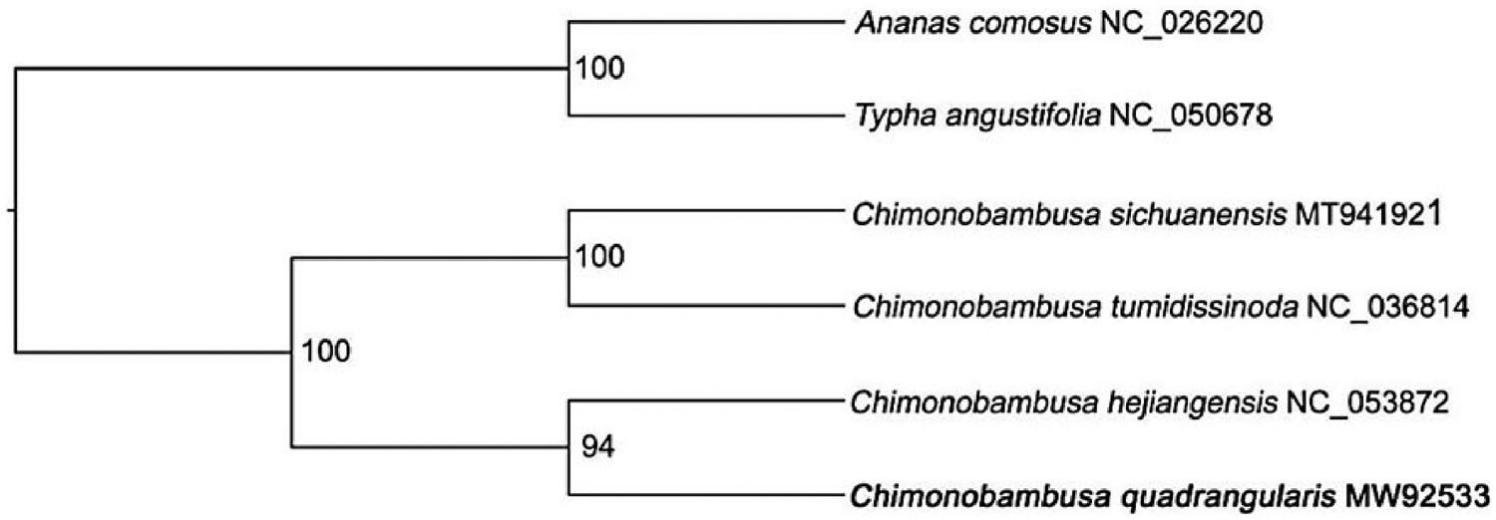
Maximum likelihood phylogenetic tree based on whole plastome sequence from 4 *Chimonobambusa* species with *Ananas comosus* (NC_026220) and *Typha angustifolia* (NC_050678) as outgroups. Bootstrap support values are shown beyond each node. *C. quadrangularis* is marked by bold font.

## Data Availability

The genome sequence data that support the findings of this study are openly available in GenBank of NCBI at https://www.ncbi.nlm.nih.gov/ under the accession No. MW928533. The associated Bio-Project, Bio-Sample and SRA numbers are PRJNA543381, SAMN19416040 and SRR14621804 respectively.
